# PDIA4, a new endoplasmic reticulum stress protein, modulates insulin resistance and inflammation in skeletal muscle

**DOI:** 10.3389/fendo.2022.1053882

**Published:** 2022-12-23

**Authors:** Chien-Hsing Lee, Chi-Fu Chiang, Fu-Huang Lin, Feng-Chih Kuo, Sheng-Chiang Su, Chia-Luen Huang, Peng-Fei Li, Jhih-Syuan Liu, Chieh-Hua Lu, Chang-Hsun Hsieh, Yi-Jen Hung, Yi-Shing Shieh

**Affiliations:** ^1^ Division of Endocrinology and Metabolism, Tri-Service General Hospital, National Defense Medical Center, Taipei, Taiwan; ^2^ Department and Graduate Institute of Biochemistry, National Defense Medical Center, Taipei, Taiwan; ^3^ School of Dentistry, National Defense Medical Center, Taipei, Taiwan; ^4^ School of Public Health, National Defense Medical Center, Taipei, Taiwan

**Keywords:** Endoplasmic reticulum, insulin resistance, metformin, PDIA4, skeletal muscle

## Abstract

**Introduction:**

Endoplasmic reticulum (ER) stress has emerged as a key player in insulin resistance (IR) progression in skeletal muscle. Recent reports revealed that ER stress-induced the expression of protein disulfide isomerase family a member 4 (PDIA4), which may be involved in IR-related diseases. A previous study showed that metformin modulated ER stress-induced IR. However, it remained unclear whether metformin alleviated IR by regulating PDIA4 expression in skeletal muscle.

**Methods:**

Herein, we used palmitate-induced IR in C2C12 cells and a high-fat diet-induced IR mouse model to document the relations between metformin, IR, and PDIA4.

**Results:**

In C2C12 cells, palmitate-induced IR increased inflammatory cytokines and PDIA4 expression. Besides, knocking down PDIA4 decreased palmitate-induced IR and inflammation in C2C12 cells. Furthermore, metformin modulated PDIA4 expression and alleviated IR both in vitro and in vivo. In addition, serum PDIA4 concentrations are associated with IR and inflammatory cytokines levels in human subjects.

**Discussion:**

Thus, this study is the first to demonstrate that PDIA4 participates in the metformin-induced effects on skeletal muscle IR and indicates that PDIA4 is a potential novel therapeutic target for directly alleviating IR.

## 1 Introduction

Obesity is a triggering factor for diabetes associated with insulin resistance (IR). IR in obesity and type 2 diabetes is manifested by decreased insulin-stimulated glucose transport and metabolism in adipocytes and skeletal muscle and by impaired suppression of hepatic glucose output (1). IR increases lipolysis, resulting in the release of free fatty acids from fat and inhibits glucose uptake by muscle cells. In addition, elevated plasma free fatty acids (FFA) are risk factors for skeletal muscle IR (2). In addition, abnormal metabolites and local inflammation lead to disruption of insulin signaling, which plays a crucial role in the development of IR (3). Skeletal muscle-induced IR phenomenon plays a key role in the development of type 2 diabetes. Study indicates that endoplasmic reticulum (ER) stress as a key factor in the progression of skeletal muscle IR (4).

When the ER protein folding capacity is overwhelmed, cells undergo a condition defined as ER stress, characterized by misfolded proteins accumulated inside the ER lumen. ER stress status, such as nutrient deprivation, hypoxia, and calcium depletion. To overcome the imbalanced ER protein-folding capacity, cells have evolved an evolutionary conserved signal transduction pathway called unfolded protein response (UPR) (5). UPR included immunoglobulin heavy chain binding protein/glucose-regulated protein 78 (BiP/GRP78) in the ER lumen, PKR-like eukaryotic initiation factor 2α kinase (PERK), inositol-requiring enzyme 1α (IRE1α), activating transcription factor 6 in ER transmembrane (ATF6), activating transcription factor 4 (ATF4), tribbles homolog 3 (TRB3), C/EBP homolog protein (CHOP), box-binding protein in X cytoplasm 1 (XBP1) (6, 7). Liver and adipose tissue of obese mice found to highly express UPR markers (8), and improve insulin sensitivity by inhibiting BiP/GRP78 expression (9). ER stress induces expression of TRB3 in skeletal muscle, which results in impairment of insulin signaling and glucose uptake. Knockdown of TRB3 significantly blunts the effects of ER stress on glucose uptake and TRB3 knockout mice are protected from high fat diet-induced IR (10). Palmitate induces increased ER stress, which in turn induces IR in skeletal muscle (11). Altogether, these results indicate that palmitate-induced ER stress is a key step in IR development in skeletal muscle. However, the detailed regulation mechanisms remain unclear.

Protein disulfide isomerases (PDIs) are expressed in many tissues (12). PDI family members include ER-resident protein (ERp) 57, ERp29, ERp44, and PDI family A member 4 (PDIA4 or ERp72) (13). PDI is a well-known multifunctional protein and has been widely involved in many diseases, including neurodegenerative diseases, metabolic diseases, osteogenesis imperfecta, cancer, infectious diseases and cardiovascular diseases (14). PDIA1 assists insulin production by regulating insulin redox in the ER (15). PDIA4 function has been shown to improve diabetes, lower blood sugar, glycated hemoglobin (HbA1C) and reactive oxygen species (ROS), and increase insulin secretion (16). Previous clinical studies have found that the serum expression of PDIA4 from patients with metabolic disease is significantly higher than that of patients without metabolic disease, suggesting that PDIA4 has the potential as a new therapeutic target for metabolic disease or IR (17). However, the role of ER stress-induced PDIA4 expression in skeletal muscle IR remains poorly understood.

Metformin is an oral antidiabetic drug from the biguanide group and plays a first-line drug for treating type 2 diabetes. Metformin lowers blood glucose by inhibiting hepatic glucose production and increasing glucose uptake in peripheral tissues, mainly skeletal muscle (18). Metformin elevates insulin receptor tyrosine kinase activity, enhances glycogen synthesis, and increases glucose transporter type 4 (GLUT4) expression (19). Metformin inhibits the expression of saturated fatty acids-induced ER stress and ER stress proteins (20). Additionally, metformin inhibits ER chaperone protein, including BiP/GRP78, PDI, and ROS production (21). Finally, metformin regulates UPR by decreasing CHOP and caspase 3 expression in human islets (22), suggesting that metformin protected islet function partially by reducing ER stress. These results imply that ER stress might be one of the therapeutic targets of metformin.

Therefore, in this study, we first explored whether ER stress-induced PDIA4 was involved in skeletal muscle IR. Next, we investigated whether metformin regulated skeletal IR by modulating ER stress and PDIA4 expression.

## 2 Materials and methods

### 2.1 Inclusion and exclusion criteria and laboratory measurements of human study subjects

The Internal Review Board of the Ethics Committee of the Tri-Service General Hospital approved this study (institutional review board approval number: 098-05-182), and all enrolled subjects provided written informed consent. The criteria for exclusion and inclusion were modified from our previous study (17). Finally, we excluded new diabetic patients and enrolled a total of 444 adults. We performed laboratory measurements (including PDIA4 levels) as described in detail in our previous study (17). We calculated the indices of β-cell function (HOMA-2β) and hepatic IR (HOMA-2IR) using HOMA Calculator v2.2.2. (23)

### 2.2 Cell culture and reagents

Mouse skeletal muscle cells (C2C12) were obtained from Bioresource Collection and Research Center (BCRC, Taiwan) and cultured in Dulbecco’s modified Eagle’s medium (DMEM) (Gibco Laboratories, Grand Island, USA) containing 10% fetal bovine serum (FBS) (Gibco Laboratories) and antibiotics (24).

### 2.3 Cell differentiation

Mouse C2C12 myoblasts were maintained in DMEM supplemented with 10% FBS. C2C12 myotubes were obtained by culturing myoblasts in DMEM containing 2% heat-inactivated horse serum for at least four days. And use passages 3-5 to perform experiments at 80% confluency. We updated the medium before the experiment. Insulin and metformin were obtained from Sigma-Aldrich (St. Louis, MO, USA) for *in vitro* experiments (25).

### 2.4 Palmitate preparation

Prepare a 40 mmol/L palmitate (C16:0; Sigma) stock solution in ethanol. Before adding cells, dilute the palmitate solution in differentiation medium containing 1% (w/v) palmitate-free BSA (Sigma) to bind palmitate to bovine serum albumin (BSA) (26). We filter sterilize the solution before adding it to the cells. The control group in this study was BSA (27).

### 2.5 Cell glucose uptake

Cellular glucose uptake was measured using a glucose uptake assay kit (Abcam, Cambridge, MA, USA). C2C12 cells were seeded at 5 × 10^4^ cells/well in 96-well plates (Corning, Inc., Corning, NY, USA) in DMEM containing 10% FBS. Use medium containing palmitate (250 μM) for 18 h. Afterwards, cells were treated with 10 nM insulin and 1 mM 2-deoxyglucose (2-DG) for 30 min. Finally, absorbance at 412 nm was measured using a BioTek Synergy HT plate reader (BioTek Instruments, Inc., Winooski, VT, USA) (28).

### 2.6 Cell viability assay

Cell viability was assessed using the MTT (3-(4,5)-dimethylthiahiazole-2-y1)-3,5-diphenytetrazolium bromide) assay. Set the MTT concentration to 5 mg/mL. Add MTT and incubate for 2 hours. The assay was terminated with DMSO. Finally measure the absorbance at 570 nm using an ELISA plate reader (29).

### 2.7 Lentiviral shRNA transfection and adenoviral infections

We obtained lentiviral-based PDIA4 and control shRNA from RNAi core of Academia Sinica, Taipei, Taiwan. We transfected the cells using PolyJet *In Vitro* DNA Transfection Reagent (SignaGen, Frederick, MD, USA) according to the manufacturer’s protocol (30).

### 2.8 Quantitative PCR analysis

We extracted total RNA using Trizol (Invitrogen, Grand Island, NY, USA). We synthesized complementary DNA (cDNA) using a SuperScript^®^ III Reverse Transcriptase kit (Invitrogen, Grand Island, NY, USA). We then performed real-time PCR for the genes of interest using SYBR green dye (Thermo, Wilmington, DE, USA) and the LightCycler^®^ 480 System (Roche). **Table 1** lists the primer sequences. We preheated the reaction mixture containing reverse transcribed cDNAs for 7 min at 95°C to activate the Taq polymerase. Next, we performed 40 PCR cycles, each consisting of a 10 s denaturation step at 95°C and a 30 s annealing step at 60°C (two-step RT-PCR) (31). Throughout the RT-PCR analysis, we confirmed product identities by melting curve analysis. The ratios of the amounts of target mRNA to the amount of the internal standard (GAPDH) mRNA was determined as an arbitrary unit.

**Table 1:** Primer sequences used for real time RT-PCR.

IL-6	Forward sequence	GACAACTTTGGCATTGTGG
	Reverse sequence	ATGCAGGGATGATGTTCTG
TNF-α	Forward sequence	GCCTCTTCTCATTCCTGCTTG
	Reverse sequence	CTGATGAGAGGGAGGCCATT
PDIA4	Forward sequence	AAGGTGGTGGTGGGAAAG
	Reverse sequence	GATGTCGTTGGCAGTAGC
CHOP	Forward sequence	CTGCCTTT CACCTTGGAGAC
	Reverse sequence	CGTTTCCTGGGGATGA-GATA
XBP1	Forward sequence	GAATGGACACGCTGGATCCT
	Reverse sequence	GCCACCAGCCTTACTCCACTC
Bip	Forward sequence	TACATCTCATGGTGGAAAGTGTCTGTTTGA
	Reverse sequence	CATCCTCCTTCTTGTCCTCCTCCTCG
ATF4	Forward sequence	GAGCTTCCTGAACAGCGAAGTG
	Reverse sequence	TGGCCACCTCCAGATAGTCATC
GAPDH	Forward sequence	CCCATCACCATCTTCCAGGAGC
	Reverse sequence	CCAGTGAGCTTCCCGTTCAGC

### 2.9 ELISA

We incubated C2C12 cells (5 × 10^4^ cells per well of a 96-well plate) with palmitate alone or combined with insulin. After incubation for 48 h, we collected the supernatants and quantified IL-6 (Catalog Number: 88-7064) and TNF-α (Catalog Number: 88-7324) using an ELISA Ready-Set-Go kit (eBioscience, San Diego, USA) according to the manufacturer’s instructions (32).

### 2.10 Western blot analysis

We harvested whole cell lysates for western blotting using RIPA buffer (1% SDS and 10 mM Tris buffer, pH 7.4) containing protease inhibitors and a phosphatase inhibitor (Thermo, Wilmington, DE, USA). We quantified proteins in the supernatants using a Pierce BCA Protein Assay Kit (Thermo, Rockford, IL, USA). We separated 30 µg of proteins on a 5%–15% gradient SDS-PAGE gel and transferred them to polyvinylidene difluoride membranes (Millipore, Bedford, MA, USA) by wet blotting using an electroblotter (Hoefer system). Next, we blocked the membranes for 1 h at 25°C with 2% BSA or 5% skim milk in tris buffered saline with tween 20 (TBST). We then incubated the membranes overnight at 4°C with appropriately diluted primary antibodies: PDIA4 (ERp72) antibody (2798 [1:1000 dilution]; Cell Signaling Technology, Danvers, MA, USA), Akt antibody (9272 [1:1000 dilution]; Cell Signaling Technology, Danvers, MA, USA), Phospho-Akt antibody (5724 [1:1000 dilution]; Cell Signaling Technology, Danvers, MA, USA), Akt antibody (9272 [1:1000 dilution]; Cell Signaling Technology, Danvers, MA, USA), Phospho-IRS-1(Ser307) antibody (2381 [1:2000 dilution]; Cell Signaling Technology, Danvers, MA, USA), IRS-1 antibody (3407 [1:2000 dilution]; Cell Signaling Technology, Danvers, MA, USA), GAPDH antibody (5174 [1:2000 dilution]; Cell Signaling Technology, Danvers, MA, USA),. After washing them in TBST three times, we incubated the membranes for 60 min at 25°C with HRP-conjugated goat anti-rabbit or anti-mouse secondary antibodies. We visualized the signal using horseradish peroxidase-conjugated secondary antibodies and the enhanced chemiluminescence assay. Finally, we determined band intensities using a UVP imaging system (33).

### 2.11 Animal model

We obtained C57BL/6J mice from the National Laboratory Animal Center, bred them in-house, and used them in accordance with the guidelines of the National Defense Medical Center of the Laboratory Animal Center (NLAC, *Taipei*, Taiwan). We fed 8 week-old male C57BL6/J mice with a chow diet (10% kcal from fat), a HFD (60% kcal from fat) (34), or a HFD with metformin (200 mg/kg, intraperitoneal) for 16 weeks (*n* = 10 in each group).

### 2.12 Insulin tolerance test

ITT is designed to determine the whole body sensitivity of insulin receptors by measuring blood glucose levels changes before and after insulin administration. For the ITT, mice fasted for 6 h received an intraperitoneal injection of human insulin (1 U kg^−1^ body weight). We collected blood samples from the tail vein before the glucose challenge and 20, 40, 60, 80, and 100 min after it. We measured serum glucose levels using a Bayer Diabetes Care analyzer (35).

### 2.13 Immunohistochemistry

We deparaffinized and rehydrated 4 μm sections of formalin-fixed, paraffin-embedded tissues and retrieved the antigen. Next, we incubated the tissue sections with hydrogen peroxide for 10 min at room temperature to quench the endogenous peroxidase. After blocking in normal goat serum, we incubated them at 4°C overnight with the primary antibodies: PDIA4 antibody (A07267 [1:200 dilution]; Biocompare, CA, USA), Phospho-AMPK antibody (2535 [1:400 dilution] Cell Signaling Technology, Danvers, MA, USA), Phospho-Akt antibody (3787 [1:400 dilution] Cell Signaling Technology, Danvers, MA, USA), Phospho-IRS-1 antibody (2381 [1:200 dilution] Cell Signaling Technology, Danvers, MA, USA), following the manufacturers’ recommendations. After washing the sections three times, we added the secondary biotinylated antibody, we incubated them for 30 min at 25°C, and added diaminobenzidine as a chromogen. Finally, we lightly counterstained these tissue sections with hematoxylin and examined them under an optical microscope. For each section examined, we counted the cells in five randomly selected fields. We analyzed the results using Image-Pro Plus (Media Cybernetics, Crofton, MA, USA) (36).

### 2.14 Statistical analysis

All statistical analyses of human data were performed using SPSS version 20.0 for Windows (SPSS Inc., Chicago, IL, USA). A P-value inferior to 0.05 was considered to indicate statistical significance. HOMA-2IR levels were divided into tertiles, with cutoff values for the tertiles of 1.59 and 2.87. We compared these tertile groups using one-way analysis of variance and the chi-square test. Relationships between PDIA4 and other variables (both anthropometric and biochemical) were analyzed by Spearman’s rank-order correlations. For *in vitro* data, represented as the means ± SEM, we used Student’s t-test to compare group pairs and one-way ANOVA to compare multiple groups. Statistical significance was evaluated with GraphPad Prism 6.01. Statistical significance was assumed at the 5% α-error level (P < 0.05).

## 3 Results

### 3.1 Human serum PDIA4 concentrations are associated with IR and inflammatory cytokines in subjects with normal glucose tolerance and impaired glucose tolerance

**Table 2** shows the characteristics of the participants separated by tertiles of homeostatic model assessment 2 (HOMA-2) IR levels. The three groups had significant differences in all anthropometric and basic biochemical values except for age, systolic blood pressure, total cholesterol, LDL, creatinine, high-sensitivity C-reactive protein (hs-CRP) and interleukin 6 (IL-6) levels. The patients in the second and third tertile of HOMA-2 IR levels had higher PDIA4 levels than those in the first tertile (P < 0.01). In addition, we evaluated the association between serum PDIA4 levels and all anthropometric values (**Table 3**). The serum PDIA4 levels showed a significant positive correlation with HOMA-2 IR and IL-6.

**Table 2:** Anthropometric and biochemical data of the participants (n = 444).

	HOMA-2IR, Tertile^a^			
	T1	T2	T3	P-value
	n = 148	n = 148	n = 148	
Age (years)	48.12 ± 14.06	49.31 ± 13.44	48.25 ± 15	0.731
BMI (kg/m^2^)	22.72 ± 3.26	24.29 ± 3.12	26.46 ± 3.81	< 0.001
WC (cm)	80.09 ± 9.61	84.41 ± 8.41	88.84 ± 10.02	< 0.001
SBP (mmHg)	126.84 ± 16.9	125.77 ± 17.1	129.25 ± 16	0.186
DBP (mmHg)	79.3 ± 10.14	79.62 ± 10.91	82.51 ± 10.33	0.015
Glucose 0’ (mg/dL)	89.58 ± 13.0	92.87 ± 11.71	101.06 ± 13.37	< 0.001
Glucose 120’ (mg/dL)	129.03 ± 36.32	127.17 ± 34.77	144.98 ± 35.97	< 0.001
Insulin 0’ (μIU/mL)	9.63 ± 4.92	12.78 ± 18.13	18.62 ± 14.29	< 0.001
Insulin 120’ (μIU/mL)	56.97 ± 35.3	82.44 ± 54.03	124.18 ± 91.14	< 0.001
HOHA-2B	83.44 ± 124.82	140.34 ± 89.9	223.12 ± 182.57	< 0.001
HOMA-2IR	1.15 ± 0.31	2.14 ± 0.34	4.76 ± 2.04	< 0.001
HbA1C (%)	5.58 ± 0.43	5.66 ± 0.35	5.89 ± 0.52	< 0.001
TC (mg/dL)	190.58 ± 32.59	192.28 ± 39	194.11 ± 34.41	0.750
LDL-C (mg/dL)	129.41 ± 29.64	128.41 ± 40.42	130.1 ± 33.03	0.957
HDL-C (mg/dL)	59.17 ± 18.88	53.04 ± 15.3	51.39 ± 12.22	0.006
TG (mg/dL)	108.79 ± 59.1	124.63 ± 76.22	149.94 ± 77.72	< 0.001
Creatinine (mg/dL)	0.86 ± 0.59	0.83 ± 0.32	0.8 ± 0.17	0.523
Uric acid (mg/dL)	5.71 ± 1.66	5.58 ± 1.35	6.18 ± 1.34	0.015
ALT (U/L)	21.83 ± 12.19	27.56 ± 21.68	39.5 ± 59.32	< 0.001
hsCRP (ng/mL)	1.08 ± 1.58	1.91 ± 5.97	2.09 ± 2.46	0.054
IL-6 (pg/mL)	1.7 ± 3.08	1.58 ± 2.12	2.11 ± 3.14	0.240
PDIA4 (ng/mL)	11.89 ± 11.64	15.12 ± 17.03	22.1 ± 19.94	< 0.001

a HOMA-2IR tertiles according to cutoff values of 1.59 and 2.87.

Data are expressed as mean ± SD.

BMI, body mass index; WC, Waist circumference; SBP, systolic blood pressure; DBP, diastolic blood pressure; HbA1C, Glycated hemoglobin; TC, total cholesterol; TG, triglycerides; HDL-C, high-density lipoprotein cholesterol; LDL-C, low-density lipoprotein cholesterol.

**Table 3:** Correlations between serum PDIA4 and clinical variables.

	Serum PDIA4	
	r	p
Age (years)	−0.045	0.349
BMI (kg/m^2^)	0.405	<0.001
WC (cm)	0.39	<0.001
SBP (mmHg)	0.098	0.041
DBP (mmHg)	0.215	<0.001
Glucose 0’ (mg/dL)	0.194	<0.001
Glucose 120’ (mg/dL)	0.213	<0.001
Insulin 0’ (μIU/mL)	0.37	<0.001
Insulin 120’ (μIU/mL)	0.325	<0.001
HOHA-2B	0.078	0.103
HOMA-2IR	0.274	<0.001
HbA1C (%)	0.176	<0.001
TC (mg/dL)	0.036	0.502
LDL-C (mg/dL)	0.137	0.051
HDL-C (mg/dL)	−0.31	<0.001
TG (mg/dL)	0.327	<0.001
Creatinine (mg/dL)	0.048	0.367
Uric acid (mg/dL)	0.244	<0.001
ALT (U/L)	0.619	<0.001
hsCRP (ng/mL)	0.253	<0.001
IL-6 (pg/mL)	0.154	0.001

BMI, body mass index; WC, Waist circumference; SBP, systolic blood pressure; DBP, diastolic blood pressure; HbA1C, Glycated hemoglobin; TC, total cholesterol; TG, triglycerides; HDL-C, high-density lipoprotein cholesterol; LDL-C, low-density lipoprotein cholesterol.

### 3.2 Palmitate can induce IR through disturbed insulin signalling, glucose uptake and increased inflammatory cytokines in C2C12 cells

**Figure 1:**
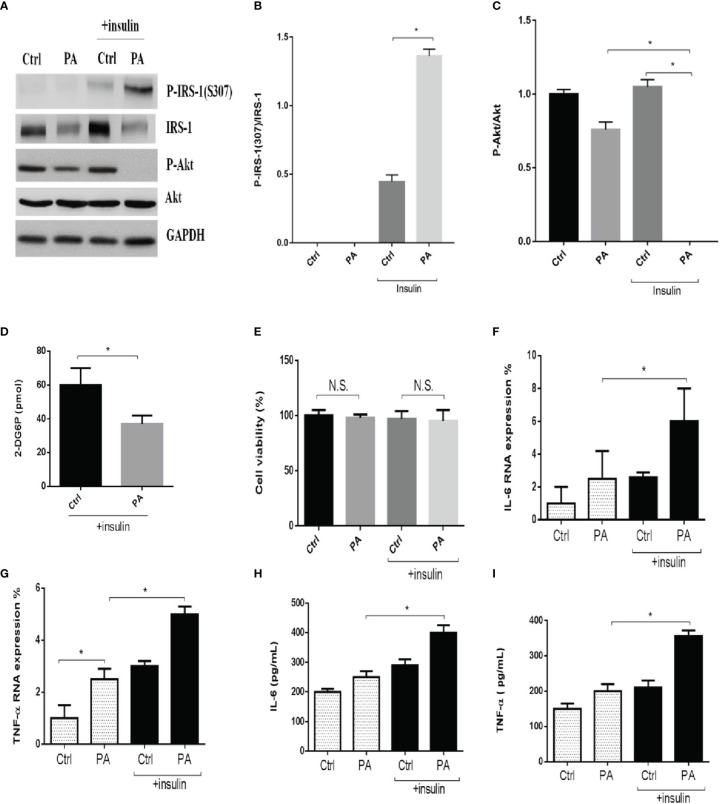
Palmitate disturbed insulin signaling and glucose uptake and increased inflammatory cytokines in C2C12 cells. The C2C12 myotubes treated with 0.6 mM of palmitate (PA) with or without 100 nM insulin, then estimated p-IRS-1(307), p-Akt by western blotting **(A-C)** and 2-Deoxyglucose (2-DG) uptake was assessed **(D)**. In addition, cell viability was measured by MTT **(E)**. The levels of **(F, G)** IL-6, TNF-α were analyzed by real-time PCR, **(H, I)** IL-6, TNF-α were analyzed by ELISA after C2C12 myotubes treated with 0.6 mM of palmitate (PA) with or without 100 nM insulin. All data are presented as the mean ± SD (n = 3 for each group); *P < 0.05. N.S, no significant.

Several studies reported that palmitate-induced IR or ER stress in obese conditions. First, we starved C2C12 myotubes from serum for 4 h and then incubated them with 0.6 mM of palmitate for another 24 h to mimic IR conditions. Next, to assess insulin action, we stimulated the cells with 100 nM insulin for a further 15 min. Then, we examined the effects of insulin signaling. We found that palmitate impaired insulin signaling. In the presence of insulin, palmitate increased the levels of phosphorylated insulin receptor substrate-1 (p-IRS-1(307), phosphorylated on serine 307)—which might contribute to IR—and decreased phosphorylated-Akt (p-Akt), while palmitate alone did not (**Figures 1A–C**). In addition, palmitate decreased glucose uptake in the presence of insulin (**Figure 1D**), but it did not affect cell viability, regardless of the presence of insulin (**Figure 1E**). Next, we examined the levels of the inflammatory cytokines IL-6 and tumor necrosis factor-α (TNF-α). In the presence of insulin, palmitate significantly increased IL-6 and TNF-α gene expression compared with palmitate alone (**Figures 1F, G**). We observed similar results for the IL-6 and TNF-α protein expressions (**Figures 1H, I**). Thus, palmitate can induce IR through disturbed insulin signaling and glucose uptake and further increase inflammatory cytokines levels in C2C12 cells.

### 3.3 Increased PDIA4 expression and ER stress markers in C2C12 cells with palmitate-induced IR

**Figure 2:**
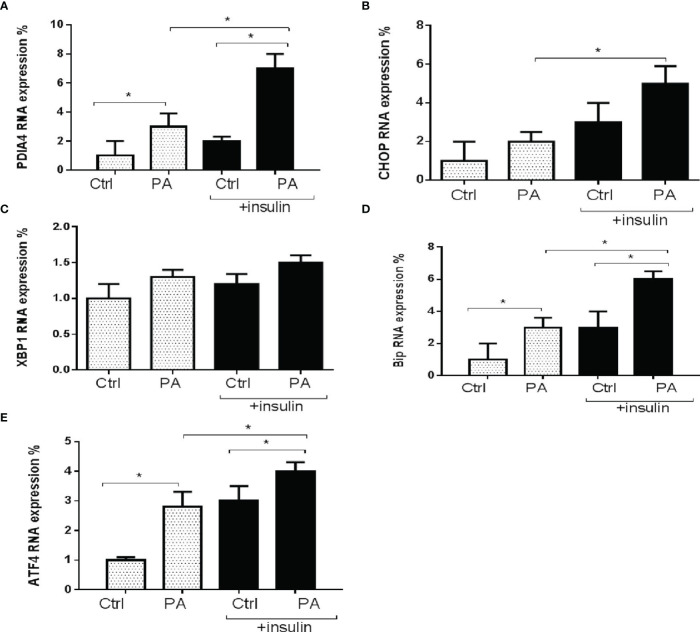
Palmitate increased PDIA4 and other ER stress markers in C2C12 cells. The levels of **(A)** PDIA4, **(B)** CHOP, **(C)** XBP1, **(D)** Bip, and **(E)** ATF4 mRNA were analyzed by real-time PCR after C2C12 myotubes treated with 0.6 mM of palmitate (PA) with or without 100 nM insulin. All data are presented as the mean ± SD (n = 3 for each group); *P < 0.05.

Next, we explored the role of PDIA4 in palmitate-induced IR in C2C12 cells. **Figure 2** shows that palmitate-treated cells had significantly higher PDIA4, BiP/GRP78, and ATF4 gene expression levels than the control group. In the presence of insulin, palmitate significantly increased PDIA4, CHOP, BiP/GRP78, and ATF4 gene expression compared with palmitate alone (**Figure 2**). We observed similar results for the protein expressions of PDIA4, BiP/GRP78, and ATF4 (**Figure S1**). Thus, cells incubated with insulin and palmitate expressed higher PDIA4 levels than those incubated with palmitate only, suggesting that PDIA4 may be an important ER stress marker in skeletal muscle IR.

### 3.4 PDIA4 knockdown decreased palmitate-induced IR and inflammation in C2C12 cells

**Figure 3:**
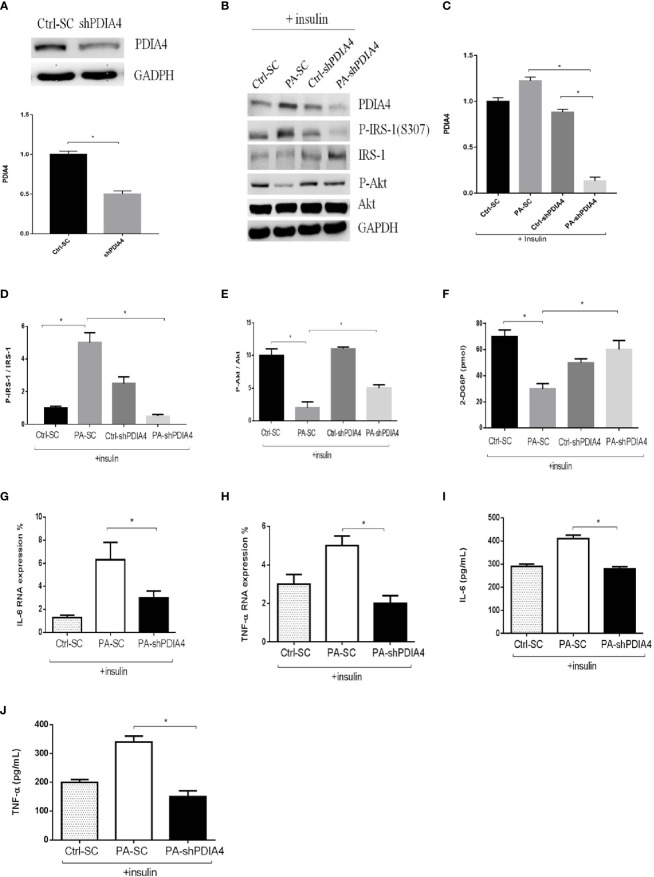
Knockdown PDIA4 inhibited palmitate induced IR, inflammatory cytokines and increased glucose uptake in C2C12 cells. **(A, B)** The protein levels of PDIA4 in C2C12 myotubes stably transfected with scrambled control (SC) or shRNA against PDIA4 (KD) were tested by western blot. **(C-E)** Knockdown PDIA4 in C2C12 myotubes, and treated with 0.6 mM of palmitate (PA) with 100 nM insulin, then estimated p-IRS-1(307), p-Akt by western blotting. **(F)** 2-Deoxyglucose (2-DG) uptake was assessed. The levels of **(G, H)** IL-6, TNF-αwere analyzed by real-time PCR, **(I, J)** IL-6, TNF-αwere analyzed by ELISA. All data are presented as the mean ± SD (n = 3 for each group); *P < 0.05.

To clarify the role of PDIA4 in IR, we inhibited PDIA4 expression in C2C12 cells with short hairpin RNA (shRNA). **Figure 3A** shows the PDIA4 knockdown efficiency. Then, we examined the effects on insulin signaling. PDIA4 knockdown cells incubated with insulin had expressed lower p-IRS-1(307) and higher IRS-1 and p-Akt levels than those incubated with palmitate alone (**Figures 3B–E**). However, in the absence of insulin, the PDIA4 knockdown decreased IRS-1 and p-Akt expression (**Figure S2**), suggesting that it restored insulin signaling. Next, we examined the glucose uptake ability. PDIA4 knockdown cells had a higher glucose uptake ability than those treated with palmitate alone (**Figure 3F**). Furthermore, knockdown PDIA4 cells had lower palmitate-induced IL-6 and TNF-α gene expressions than non-knockdown cells (**Figures 3G, H**). Besides, we observed similar results for the protein expressions of IL-6 and TNF-α (**Figures 3I, J**). Overall, knocking down PDIA4 mitigated IR and decreased inflammatory cytokines expression in C2C12 cells.

### 3.5 Metformin modulated PDIA4 expression and palmitate-induced IR in C2C12 cells

**Figure 4:**
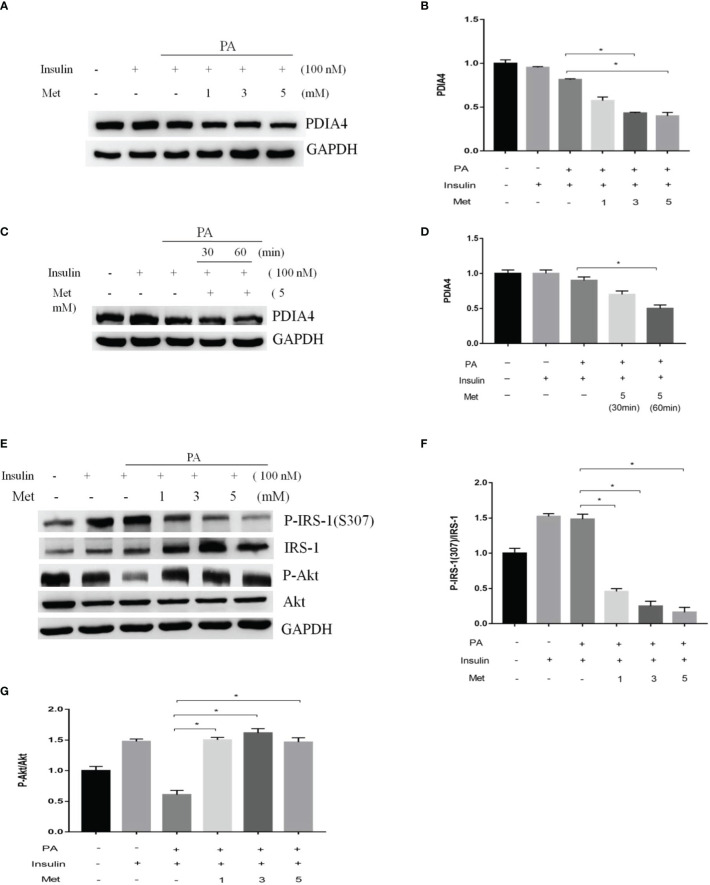
Metformin modulated PDIA4 expression and palmitate-induced IR in C2C12 cells. **(A, B)** The C2C12 myotubes treated 1,3,5 mM metformin with 100 nM insulin, then estimated PDIA4 expression by western blotting. **(C, D)** The C2C12 myotubes treated 5 mM metformin with palmitate and insulin, then estimated PDIA4 expression by western blotting. **(E–G)** The C2C12 myotubes treated 1,3,5 mM metformin with palmitate and insulin, then estimated p-IRS-1(307), p-Akt by western blotting. All data are presented as the mean ± SD (n = 3 for each group). *P < 0.05.

**Figure 5:**
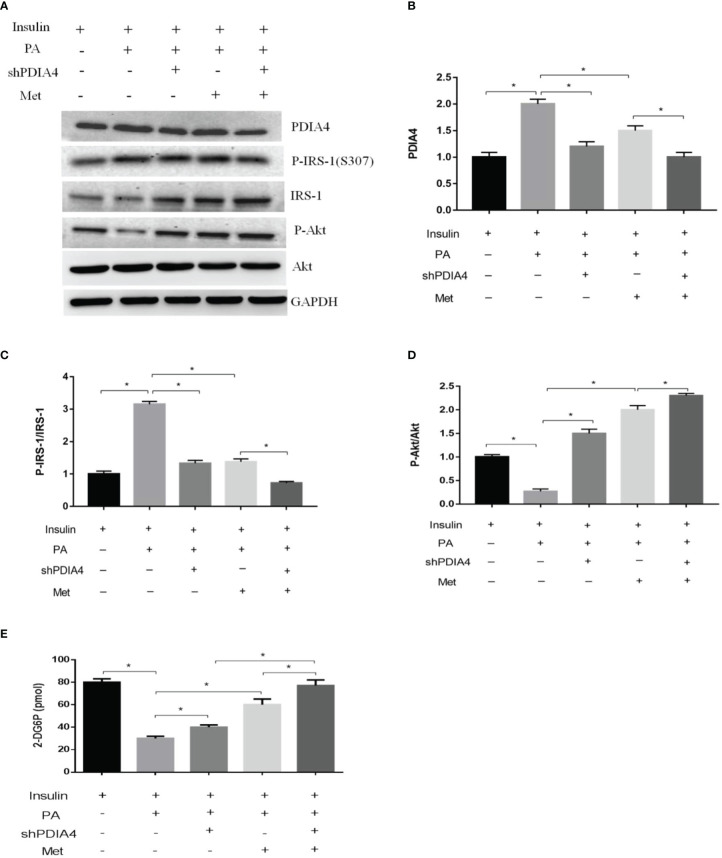
Metformin and PDIA4 knockdown in palmitate-induced IR and glucose uptake in C2C12 cells. **(A)** The PDIA4 knockdown of C2C12 myotubes treated metformin with palmitate and insulin, then estimated PDIA4, p-IRS-1(307), p-Akt by western blotting. **(B-D)** Quantization from western blot PDIA4, p-IRS-1(307), p-Akt. **(E)** 2-Deoxyglucose (2-DG) uptake was assessed knockdown PDIA4 in C2C12 myotubes treated metformin with palmitate and insulin. All data are presented as the mean ± SD (n = 3 for each group). *P < 0.05.

Metformin is a common clinical treatment for IR (37). Thus, we explored whether metformin mitigated IR through PDIA4. We treated C2C12 with 1, 3, or 5 mM metformin after incubating them with palmitate and insulin. Metformin significantly decreased PDIA4 expression in the doses of 3 and 5 mM (**Figures 4A, B**) and on the time of 60 minutes (**Figures 4C, D**). Furthermore, in cells treated with insulin and palmitate, metformin decreased p-IRS-1(307) and increased IRS-1 and p-Akt expression (**Figures 4E–G**). Next, we assessed the effect of metformin on IR through PDIA4 by treating PDIA4 knockdown C2C12 cells with metformin after culturing them with palmitate and insulin. The result showed PDIA4 knockdown, and metformin have mild additive effects on the suppression of PDIA4 expression (**Figures 5A, B**). PDIA4 knockdown cells were more sensitive to the metformin-induced decrease in p-IRS-1(307) and increase in IRS-1 and p-Akt expression than normal C2C12 cells (**Figures 5A, C, D**). Moreover, the metformin-induced increase in glucose uptake was higher in the PDIA4 knockdown cells than in normal C2C12 cells (**Figure 5E**). Collectively, these results suggested that metformin can strengthen the inhibition of PDIA4 expression and improve IR and glucose uptake.

### 3.6 Metformin decreased PDIA4 expression and mitigated IR in a high-fat diet-induced mouse obesity model

**Figure 6:**
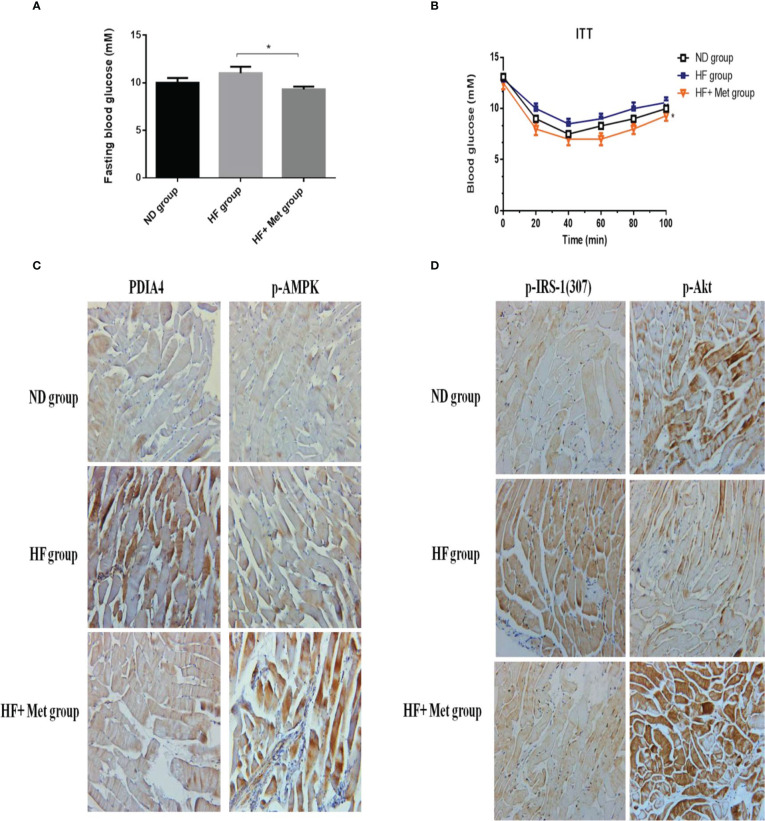
Metformin decreased PDIA4 and improved IR in high-fat diet induced obesity mice model. Administered metformin (200mg/kg) through intraperitoneal injection into high fat diet (HFD, 60% kcals from fat) induced obesity in C57BL6/J mice, and a chow diet (10% kcals from fat) as control group, and then examined **(A, B)** basal blood glucose, and insulin tolerance. **(C, D)** The mice soleus muscle tissues were examined PDIA4, P-AMPK, P-IRS-1(307), and P-Akt by immunohistochemistry, 20X. Representative data were shown from experiments independently performed at least three times. *P < 0.05.

**Figure 7:**
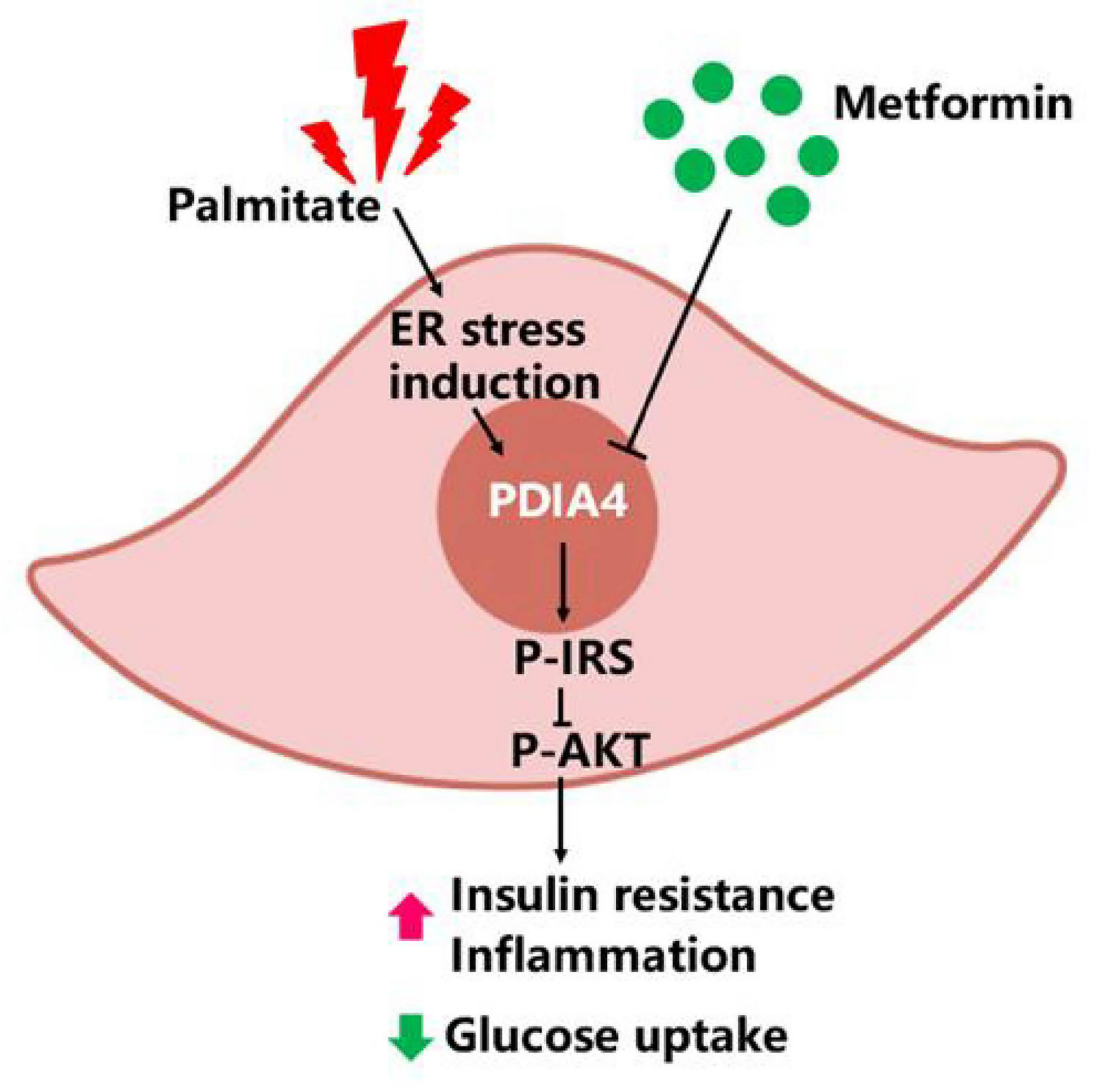
PDIA4 in skeletal muscle insulin resistance (IR). Palmitate induced IR increased PDIA4 and inflammatory cytokines, and then impaired insulin signaling and reduced glucose uptake. PDIA4 knockdown decreased inflammatory cytokines and improved IR. Furthermore, PDIA4 was involved in metformin improved skeletal muscle IR and suggested that PDIA4 may be a novel therapeutic target for directly alleviating IR.

HFDs affect skeletal muscle function and cause IR in mice (38). Metformin inhibits the mitochondrial respiratory chain, activating the AMP-activated protein kinase (AMPK) and enhancing insulin sensitivity (39). To establish a causal relationship between metformin, obesity, and inflammation in an animal model, we administered metformin (200 mg/kg) through intraperitoneal injection to C57BL6/J mice with HFD-induced obesity (HFD, 60% kcal from fat) and mice fed a chow diet (10% kcal from fat) as a control group. As expected, metformin improved basal blood glucose levels in HFD mice (**Figure 6A**). Likewise, metformin-treated mice exhibited significantly lower glucose concentrations than untreated mice in the insulin tolerance test (ITT) (**Figure 6B**). Next, we examined the expression levels of p-IRS-1(307), p-Akt, PDIA4, and p-AMPK by immunohistochemistry in mice soleus muscle tissues. As expected, metformin decreased p-IRS-1(307) and PDIA4 expression and increased p-Akt and p-AMPK expression in HFD mice (**Figures 6C, D**), suggesting that metformin improved skeletal muscle IR may by increasing AMPK and decreasing PDIA4 expression. Based on the above results, palmitate-induced IR increased PDIA4 and inflammatory cytokines expression, impaired insulin signaling, and reduced glucose uptake. Meanwhile, the PDIA4 knockdown decreased inflammatory cytokines levels and mitigated IR. Furthermore, PDIA4 participates in the metformin-induced effects on skeletal muscle IR (**Figure 7**).

## 4 Discussion

Chronically elevated FFA levels may contribute to IR progression. FFA and their metabolites can also interfere with insulin signaling and inhibit insulin-stimulated glucose uptake and glycogen synthesis (40). Excessive FFA levels in the blood increase lipid metabolites accumulation in skeletal muscle, leading to IR (41). The skeletal muscle is the primary site for insulin-stimulated glucose disposal and is sensitive to the insulin action mediated by elevated fatty acid availability in the human body (42). In this study, we utilized a well-established IR model by treating C2C12 myotubes with palmitate and insulin (43). Some studies showed that palmitate increased serine phosphorylation of IRS-1 and apoptosis in rat insulinoma cells and decreased Akt phosphorylation and glucose uptake (43, 44). Meanwhile, metformin attenuated the palmitate-induced increase in apoptosis and BiP/GRP78 and PDI expression (44).

In addition, c-Jun N-terminal kinase (JNK)-dependent serine (307) phosphorylation of IRS-1 is a key link between ER stress and IR (45). Chronic inflammation, which is related to IR and obesity, can be initiated by excessive lipid deposition in muscle, fat, and liver (46). The previous report showed palmitate-induced C/EBP homologous protein activation leads to NF-κB-mediated increase in β-site amyloid precursor protein cleaving enzyme 1 activity and amyloid beta genesis in neuroblastoma cells (47). Moreover, Tae Woo Jung et al. revealed palmitate-induced aggravation of insulin signaling markers, such as IRS-1 and Akt phosphorylation, and inflammatory markers, such as NF-κB and IκB phosphorylation in C2C12 myocytes (48) . In our study, palmitate can induce IR through disturbed insulin signaling and glucose uptake and further increase inflammatory cytokines levels in C2C12 cells. The possible mechanisms by which palmitate induces inflammation in C2C12 cells, maybe through activating the NF-κB pathway. Several studies have demonstrated the role of ER stress to mediate palmitate-induced IR in muscle cells. These have shown that direct exposure of human primary myotubes, C2C12 myotubes, or L6 myotubes to palmitate can induce ER stress (49). Another study showed the palmitate-induced pathway driving the migration of the cancer cells through the loss of desmoplakin mediated by activation of the IRE1-XBP1 pathway and zinc finger E-box binding homeobox transcription factors (50). Palmitate disrupts erythropoietin production by activating the transcription factor ATF4, which is involved in the UPR (51). Besides, palmitate and insulin also increased ATF4, CHOP, XBP1, and BiP/GRP78 expressions in our skeletal muscle IR model. However, little is known about the link between FFA metabolism and ER stress-related effects on PDIA4 and IR. Our study showed that palmitate and insulin increased PDIA4, inflammatory cytokines, and p-IRS-1 levels and decreased p-Akt levels and 2-DG uptake in a skeletal muscle IR cell model. Furthermore, mice with HFD-induced IR had elevated expression levels of p-IRS-1(307) and PDIA4 in soleus muscle tissues.

The PDI family plays a role in mammalian development and in various diseases. The nine human PDI family members contain one to three CGHC active sites (52). Among them, only PDIA4 has three CGHC motifs. Most of the PDIs have an ER retention motif (53) . However, increasing data show that PDIs have also been found in locations outside the ER, including the cell surface, nucleus, cytoplasm, and extracellular space (54) and plasma of different cell types. Accordingly, PDI is a multifunctional protein including in a variety of redox-related intracellular and extracellular events and functions such as other ER chaperones (55, 56). Previous studies revealed BiP/GRP78, an ER chaperone, has been detected in cell membranes, where it acts as a multireceptor and signal receptor transducer and mediates other functions (57). Bip/GRP78 was released into culture medium from challenged cells to induce ER stress. A soluble part of the BiP/GRP78 protein can be detected in circulation, probably due to active secretion rather than simply a result of cell necrosis or apoptosis (58, 59). Moreover, the circulating BiP/GRP78 levels are significantly increased in people with DM, obesity, and its associated metabolic alterations (60) . Recent study indicated that PDIA4 was distributed in the nuclei, cytosol, membrane, mitochondria, and ER of Min6 β-cells and serum PDIA4 also went up with diabetes development in HFD-fed B6 mice, and diabetic patients (16). Our previous report revealed that subjects with metabolic syndrome had significantly higher serum PDIA4 levels than those without metabolic syndrome. Furthermore, the individuals in the highest PDIA4 tertile had significantly higher waist circumference, blood pressure, fasting glucose concentration, and serum triglycerides than those in the lowest tertile (17). In this study, we used our previous data set but excluded new diabetic patients. We found that serum PDIA4 levels were associated with IR and inflammatory cytokines levels in subjects with normal or impaired glucose tolerance. These results were consistent with our recent report (61). Moreover, individuals in the second and third tertile of HOMA-2 IR levels had higher PDIA4 levels than those in the first tertile. Furthermore, in this study’s population, the serum PDIA4 levels had a significant positive correlation with HOMA-2 IR and IL-6 levels.

PDI regulates biological processes, such as protein folding, signal transmission, and cell communication, involving the interaction between PDI and substrate proteins (55, 62). Recently, PDIA1 has been characterized as a molecular chaperone to activate estrogen receptor *via* stabilizing the receptor (55, 63). Besides, ablating PDIA4 reduced the symptoms of diabetes, such as elevated blood sugar and HbA1C levels, in diabetic mice (16). However, the role of PDIA4 in skeletal muscle IR remained unknown. Our PDIA4 knockdown experiment results showed that PDIA4 participates in skeletal muscle IR. Indeed, knocking down PDIA4 decreased inflammatory cytokines and p-IRS-1 levels and increased Akt phosphorylation and 2-DG uptake in palmitate and insulin-treated C2C12 myotubes. In summary, knocking down PDIA4 expression mitigated palmitate-induced IR, glucose uptake and inflammation in C2C12 cells. IRS-1 is a substrate of the insulin receptor and the phosphorylation of serine residues in IRS-1 plays a critical role in the insulin-stimulated signaling pathway. The possible mechanisms of PDIA4 action in insulin signaling and GLUT4-mediate glucose uptake might be through the interaction between PDIA4 and IRS-1, Akt substrate proteins, or insulin receptor then disturbed the insulin signaling and glucose management in palmitate-treated C2C12 cells subsequently.

Metformin is an effective hypoglycemic drug (64) and a widely used insulin sensitizer that lowers blood glucose concentrations by decreasing hepatic glucose production and increasing glucose disposal in skeletal muscle. However, the molecular mechanism of metformin action is not well understood. Previous reports showed that metformin regulated systemic glycemia by stimulating AMPK in the liver and skeletal muscle and enhanced insulin signaling in skeletal muscle IR by increasing IRS-1 and Akt activity (19). The JNK-dependent phosphorylation of the serine 307 of IRS-1 is a key link between ER stress and IR. In rat insulinoma cells, metformin attenuated ER stress, IRS-1 phosphorylation, and apoptosis (44). Other studies indicated that metformin suppresses ER stress through the AMPK- PI3K-c-Jun NH2 pathway in NIT-1 cells (65), reduces ER stress-induced brain injury, and inhibits apoptosis by regulating the protein kinase R (PKR)-like ER kinase (PERK)-eIF2α-ATF4-CHOP pathway (66). Recently, our report revealed the clinical insulin sensitizer metformin modulates PDIA4 and adiponectin expression and improves obesity-associated conditions in both *in vitro* adipocytes and *in vivo* mouse models (67). However, the role of ER stress-related PDIA4 in the effect of metformin on skeletal muscle IR remained elusive. Our results demonstrated that metformin decreased PDIA4 expression in a time- and concentration-dependent manner, decreased p-IRS-1(307), and increased p-Akt in IR skeletal muscle. Moreover, our data revealed that knocking down PDIA4 potentiated the additive effect of metformin on IRS-1 and Akt phosphorylation and 2-DG uptake. However, it is known that metformin does decrease ER stress. Our study revealed that metformin may alleviate skeletal muscle ER and at least partially through inhibiting PDIA4 expression.

Skeletal muscle is the major site for insulin-stimulated glucose disposal, and muscle IR has many adverse health outcomes. Feeding rodents with an HFD for several weeks or months induces IR (68). Male C57BL/6J mice on an HFD providing 60 kcal energy from fat for nine weeks display impaired insulin sensitivity and chronic inflammation (69). Metformin alleviates skeletal muscle IR in an HFD-induced IR rat model (70). In our animal experiments, we observed substantial body weight differences between the HFD and HFD with metformin groups (data not shown), indicating that metformin had beneficial effects on body weight gain without affecting daily food intake (data not shown). Besides, the ITT experiment showed that metformin improved IR in HFD-fed mice. One mechanism mediating IR may involve the phosphorylation of serine residues in IRS-1, preventing IRS-1 from activating downstream PI3K-Akt-dependent pathways (71). IRS-1 is a substrate of the insulin receptor and plays a central role in the insulin-stimulated signal transduction pathway. Some studies showed that metformin does not influence β-cell secretion of insulin or IRS-1 in skeletal muscle, whereas one study reported decreased IRS-1 in soleus muscle after metformin treatment (72). In our animal experiments, metformin decreased p-IRS-1 and increased p-Akt in the soleus muscle. A previous report showed that the metformin-induced AMPK activation decreases cardiac injury during ER stress by preventing CHOP expression in C57BL/6 mice (73). Our *in vivo* animal experiments demonstrated that metformin increased AMPK and decreased PDIA4 expression in the soleus muscle. However, it is known that metformin does increase AMPK activation, the decreased PDIA4 may still be an indirect effect of the decrease in ER stress.

The present study has several limitations. First, our human clinical data only showed an association between PDIA4 and IR and inflammatory cytokines. Determining the effect of metformin on PDIA4 concentrations requires further studies. Second, an animal model with PDIA4 conditional knockout in skeletal muscle could confirm the direct causal relationship between metformin, PDIA4, and skeletal muscle IR. Third, in this study we only used genetic inhibition of PDIA4 in *in vitro* cell model. In the future, we will find and use PDIA4 specific inhibitor as pharmacological inhibition for future studies. Fourth, physiological insulin is well under 1nM, but the concentration of insulin added in this cell experiment is 100 nM. The correlation between the results of the cell experiment and clinical application still needs more research to be carried out.

In conclusion the study is the first to explore the role of PDIA4 in IR skeletal muscle. We elucidated that palmitate-induced IR increased PDIA4 and inflammatory cytokines expression, impaired insulin signaling, and reduced glucose uptake in skeletal muscle cells. Conversely, knocking down PDIA4 decreased inflammatory cytokines expression and mitigated skeletal muscle IR. Furthermore, we demonstrated that metformin may mitigate skeletal muscle IR and at least partially through inhibiting PDIA4 expression. More importantly, our results suggest that PDIA4 is a novel therapeutic target for directly alleviating skeletal muscle IR. In addition,

## Data availability statement

The raw data supporting the conclusions of this article will be made available by the authors, without undue reservation.

## Ethics statement

Tri-Service General Hospital approved this study (institutional review board approval number: 098-05-182). The patients/participants provided their written informed consent to participate in this study. The animal study was reviewed and approved by National Defense Medical Center of the Laboratory Animal Center (NLAC, Taipei, Taiwan).

## Author contributions

C-HsL, C-FC, Y-SS contributed to conception and design of the study. F-HL, F-CK, S-CS organized the database. C-LH, P-FL, J-SL performed the statistical analysis. C-FC wrote the first draft of the manuscript. C-HsL, C-HuL, C-HH, and Y-JH wrote sections of the manuscript. All authors contributed to the article and approved the submitted version.
