# Supplementary material for: PDIA4, a new endoplasmic reticulum stress protein, modulates insulin resistance and inflammation in skeletal muscle
Source: Front Endocrinol (Lausanne). 2022 Dec 23;13:1053882. doi: 10.3389/fendo.2022.1053882 (PMC9816868; doi:10.3389/fendo.2022.1053882)
Supplement: Supplementary file 1 [file DataSheet_1.docx]

Supplementary Figure


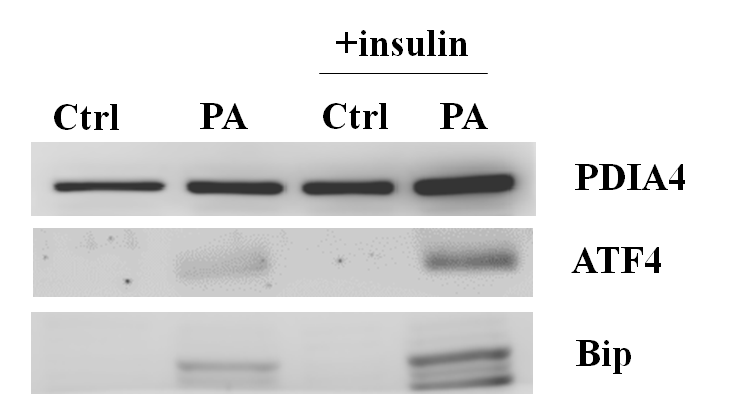


**Supplementary Figure 1. Palmitate increased PDIA4, other ER stress markers in C2C12 cells.** The C2C12 myotubes treated with 0.6 mM of palmitate (PA) with or without 100 nM insulin, then estimated ER stress markers PDIA4, ATF4, and Bip by western blotting. All data are presented as the mean ± SD (n = 3 for each group).


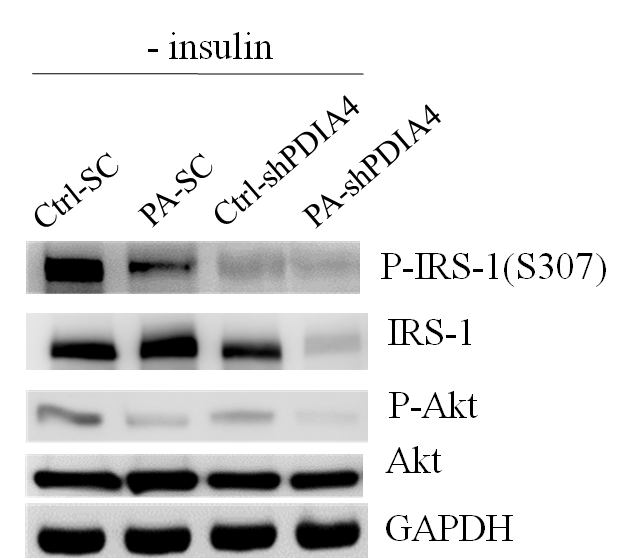


**Supplementary Figure 2. Knockdown PDIA4 inhibited palmitate induced IR in C2C12 cells.** Knockdown PDIA4 in C2C12 myotubes , and treated with 0.6 mM of palmitate (PA) without 100 nM insulin, then estimated p-IRS-1(307), p-Akt by western blotting. All data are presented as the mean ± SD (n = 3 for each group).
